# Central Cord Syndrome: Surgical vs Conservative Management

**DOI:** 10.7759/cureus.115812

**Published:** 2026-09-05

**Authors:** Georgios Piperas, Ioannis S Benetos, Angelos Kaspiris, Thomai Pipera, Evgenia Stagaki, Panagiotis Fragkos, John Vlamis

**Affiliations:** 1 First Department of Orthopedic Surgery, KAT Hospital, Athens, GRC; 2 Third Department of Orthopedics, University of Athens, KAT Hospital, Athens, GRC; 3 Sixth Department of Orthopedic Surgery, KAT Hospital, Athens, GRC; 4 Department of Obstetrics and Gynecology, Venizeleio Hospital, Heraklion, GRC

**Keywords:** central cord syndrome, cervical spine, conservative management, spinal cord injury, surgical decompression

## Abstract

Central cord syndrome (CCS) represents the most prevalent type of incomplete traumatic spinal cord injury and is characterized by disproportionately greater motor impairment in the upper extremities compared to the lower extremities. Despite its high prevalence, the optimal therapeutic approach remains controversial, particularly regarding the choice between surgical and conservative management. A scoping review of the literature was conducted using PubMed, Scopus, ScienceDirect, and Elsevier databases, including studies published between 1954 and 2026. After the application of predefined inclusion and exclusion criteria, 20 studies were included in the final analysis. The available evidence suggests that conservative management remains a safe and effective option in selected patients, particularly in the absence of spinal instability or significant spinal cord compression and in cases with mild neurological deficits demonstrating early improvement. In contrast, surgical decompression appears to provide superior neurological and functional outcomes in patients with mechanical compression, spinal instability, or progressive neurological deterioration. Early surgical intervention has been increasingly associated with improved recovery, although the literature remains heterogeneous. In conclusion, the management of CCS should be individualized and guided by clinical and imaging findings. While conservative treatment remains appropriate in selected cases, current evidence supports an expanding role for early surgical intervention in appropriately selected patients.

## Introduction and background

Central cord syndrome (CCS) is the most common form of incomplete traumatic spinal cord injury and represents a distinct clinical entity characterized by disproportionately greater motor impairment in the upper extremities compared to the lower limbs [1]. The syndrome was first systematically described in 1954 by Schneider et al., who defined it as a condition characterized by greater motor weakness in the upper extremities than in the lower extremities, accompanied by variable sensory deficits and frequently bladder dysfunction [2,3]. This classical definition remains fundamental in contemporary clinical practice [2].

Earlier observations of similar neurological patterns had been reported in the late 19th century by Sir William Thorburn; however, Schneider was the first to clearly define the syndrome and propose its underlying pathophysiological mechanism [1]. He attributed CCS primarily to hyperextension injuries occurring in the setting of preexisting cervical spondylosis, leading to central spinal cord damage. His work also described the characteristic pattern of neurological recovery, typically beginning in the lower extremities and progressing to the upper limbs [3].

Epidemiologically, CCS accounts for approximately 9% of adult spinal cord injuries and represents the most frequent incomplete spinal cord injury syndrome [4]. The incidence of CCS has increased in recent decades, largely due to the aging population and the higher prevalence of degenerative cervical spine conditions [1]. The condition demonstrates a bimodal distribution [5,6]. In older individuals, CCS typically results from low-energy trauma, such as falls, in the presence of cervical spinal stenosis caused by degenerative changes. In younger populations, it is more commonly associated with high-energy mechanisms, including motor vehicle accidents and sports-related injuries [4].

The pathophysiology of CCS is complex and multifactorial. While traditionally attributed to the somatotopic organization of corticospinal tracts, more recent evidence highlights the role of vascular compromise, microhemorrhage, and secondary inflammatory processes. The central location of the injury results in disproportionate involvement of upper limb function, particularly fine motor control [1].

Despite its relatively favorable natural history, with many patients demonstrating spontaneous neurological improvement, CCS remains a challenging clinical condition in terms of management. Historically, conservative treatment was considered the standard approach. However, advances in surgical techniques and improved understanding of spinal cord injury mechanisms have led to increasing interest in early surgical decompression [7].

Nevertheless, the optimal management strategy remains controversial. Conflicting evidence exists regarding the relative benefits of conservative vs surgical treatment, particularly in patients without clear spinal instability or mechanical compression. The aim of this study is to systematically review and critically evaluate the available literature on the management of CCS, with emphasis on comparing conservative and surgical approaches and identifying key prognostic factors influencing clinical outcomes [7].

## Review

Materials and methods

This study was designed as a scoping review to evaluate the existing literature on the management of CCS. A comprehensive search was conducted using PubMed, Scopus, ScienceDirect, and Elsevier databases, including studies published between 1954 and 2026. The search strategy included combinations of keywords such as “central cord syndrome”, “spinal cord injury”, “conservative management”, “surgical treatment”, “prognosis”, and “cervical spine”. Titles and abstracts were initially screened to assess relevance. Full-text articles were subsequently reviewed based on predefined inclusion and exclusion criteria.

Studies were included if they involved human subjects with CCS and evaluated conservative and/or surgical treatment outcomes. Only English-language publications with available abstracts were considered. Studies were excluded if they involved animal models, in vitro data, non-English publications, or case reports with small sample sizes.

A total of 132 studies were identified. After removal of duplicates (n = 34) and exclusion based on title and abstract screening (n = 98), 35 full-text articles were assessed. Of these, 15 were excluded for the following reasons: animal studies/in vitro studies (n = 5), studies in languages other than English (n = 3), research protocols (n = 4), case reports with small sample sizes (n = 2), and studies not relevant to therapeutic comparison (n = 1). Ultimately, 20 studies were included in the final analysis (Figure 1).

**Figure 1:**
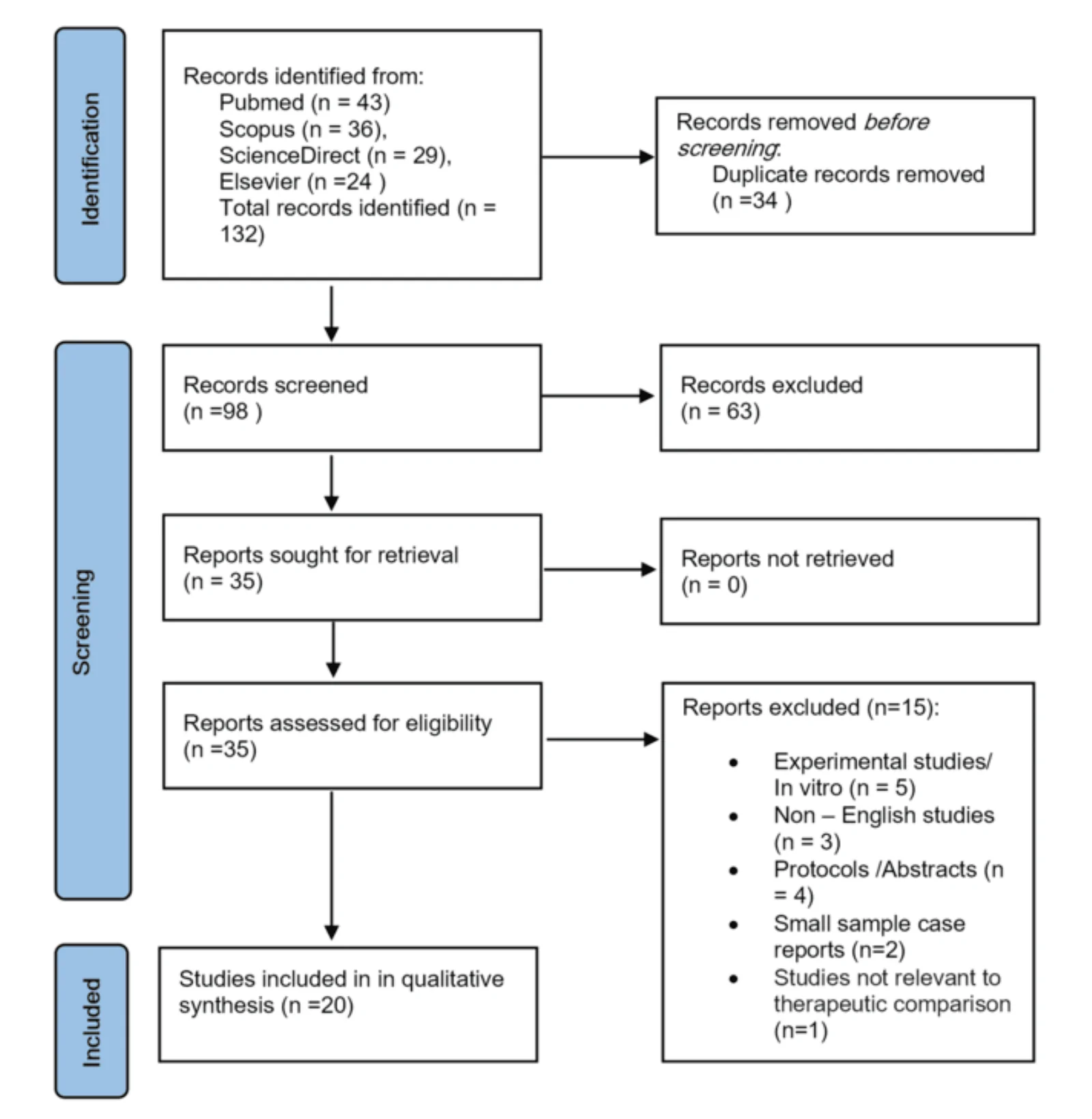
Study flowchart.

Results 

The included studies demonstrated significant heterogeneity in terms of study design, patient populations, and outcome measures. Most studies were retrospective or observational, with limited high-quality randomized data available.

Conservative management was found to be effective in selected patients, particularly those without spinal instability or significant cord compression. Patients with mild neurological deficits often showed substantial spontaneous recovery. Improvement typically followed a characteristic sequence, beginning in the lower extremities and progressing to upper limb function. Functional outcomes such as ambulation and bladder control were frequently regained [8]. Surgical management was primarily indicated in patients with mechanical compression, spinal instability, or progressive neurological deterioration. Surgical decompression was associated with improved neurological outcomes, particularly in patients with more severe deficits. Both anterior and posterior approaches were used depending on the underlying pathology [9,10].

The timing of surgical intervention emerged as an important factor. Early decompression was associated with improved outcomes in several studies, although findings across the literature were not entirely consistent [1,7,11-13]. Prognostic factors identified included initial neurological status, age, and imaging findings. Patients with less severe initial deficits and younger age generally demonstrated better outcomes [14,15].

To facilitate a clearer understanding of the differences between conservative and surgical approaches, a comparative summary of their key characteristics, indications, and outcomes is presented in Table 1.

**Table 1:** Comparison of conservative and surgical management in CCS. CCS, central cord syndrome

Parameter	Conservative management	Surgical management
Primary indications	Mild neurological deficits, absence of spinal instability, and no significant cord compression	Mechanical compression, spinal instability, or progressive neurological deterioration
Mechanism of action	Supports spontaneous neurological recovery through immobilization, hemodynamic optimization, and rehabilitation	Direct decompression of the spinal cord with or without stabilization
Advantages	Avoids operative and anesthesia-related risks; appropriate for stable patients with early neurological improvement	Provides immediate decompression, may accelerate recovery, and limits ongoing structural injury
Disadvantages	Potentially slower recovery and risk of incomplete improvement in patients with persistent compression or more severe deficits	Risk of perioperative complications, anesthesia-related morbidity, and approach-specific complications
Neurological outcomes	Generally favorable in carefully selected mild cases, particularly when early clinical improvement is observed	More favorable in patients with moderate-to-severe deficits, ongoing compression, or progressive neurological decline
Timing considerations	Initiated immediately after injury as part of acute supportive management	Early decompression, usually within 24-72 hours, may improve neurological and functional outcomes in selected patients
Ideal patient profile	Stable cervical spine, mild deficits, no significant radiological compression, and early spontaneous improvement	Identifiable structural pathology, worsening neurological status, instability, or persistent spinal cord compression

Discussion 

The management of CCS remains a subject of ongoing debate, reflecting the heterogeneity of the condition and the variability in clinical presentation, imaging findings, and patient characteristics. The present review highlights that the decision between conservative and surgical management cannot be approached uniformly but rather requires a tailored strategy based on individual patient factors.

Conservative management has historically constituted the mainstay of treatment for CCS, largely due to the observation that a substantial proportion of patients experience spontaneous neurological recovery. This approach typically includes immobilization, optimization of spinal cord perfusion, and early initiation of rehabilitation [16,17]. Evidence suggests that patients without spinal instability or significant mechanical compression, particularly those presenting with mild neurological deficits, often demonstrate favorable outcomes with conservative treatment. The pattern of recovery in these patients is characteristic, with early improvement in lower extremity function followed by gradual restoration of upper limb strength and fine motor control. Functional outcomes such as ambulation and bladder control are frequently regained, supporting the safety and effectiveness of nonoperative management in carefully selected cases [18].

Conservative management of CCS encompasses a combination of acute supportive measures, pharmacological interventions, and structured rehabilitation strategies aimed at minimizing secondary spinal cord injury and promoting functional recovery. In the acute phase, management follows the principles of traumatic spinal cord injury care, including spinal immobilization, maintenance of adequate oxygenation, and optimization of hemodynamic parameters. In particular, maintenance of mean arterial pressure at levels sufficient to ensure adequate spinal cord perfusion has been emphasized as a critical component in preventing secondary ischemic injury [16,19].

Pharmacological management plays a supportive but important role in the acute setting. The use of high-dose corticosteroids, particularly methylprednisolone, has historically been proposed based on studies suggesting a potential neuroprotective effect when administered within the first hours after injury. However, subsequent analyses have raised concerns regarding limited clinical benefit and increased risk of complications, such as infections and gastrointestinal bleeding. As a result, the routine use of corticosteroids remains controversial, and current guidelines do not universally recommend their administration, although they may be considered in selected cases [1,11]. Additional pharmacological strategies include adequate pain management, which is essential given the high prevalence of neuropathic and musculoskeletal pain in these patients. Analgesics, including nonsteroidal anti-inflammatory drugs and neuropathic pain agents, may be used depending on the clinical presentation [20]. Furthermore, emerging neuroprotective agents, such as riluzole, have been investigated for their potential to reduce excitotoxicity and limit secondary neuronal damage. Although early results are promising, their role in clinical practice has not yet been fully established [21,22].

Beyond pharmacological treatment, conservative management includes early mobilization when feasible and comprehensive rehabilitation programs focusing on physical therapy, occupational therapy, and functional training. These interventions aim to enhance neuroplasticity, improve motor function, and facilitate recovery of activities of daily living. The rationale behind conservative management is based on the intrinsic capacity for neurological recovery observed in CCS, particularly in cases without ongoing mechanical compression [1]. The gradual improvement observed in many patients is thought to reflect the resolution of edema, restoration of spinal cord perfusion, and adaptive neuroplastic mechanisms. However, the effectiveness of conservative treatment is highly dependent on appropriate patient selection and remains limited in cases where structural pathology contributes to ongoing spinal cord compromise [23,24].

Despite these favorable outcomes, conservative management is not universally appropriate. Patients with more severe neurological deficits, lack of early improvement, or radiological evidence of ongoing spinal cord compression may not benefit from a nonoperative approach. In such cases, delayed intervention may result in prolonged disability or incomplete neurological recovery, highlighting the limitations of purely conservative treatment strategies [1,25].

In contrast, surgical management has gained increasing attention in recent years, driven by advances in imaging, surgical techniques, and understanding of secondary spinal cord injury mechanisms [11,18]. The primary goal of surgical intervention is to achieve adequate decompression of the spinal cord and, when necessary, stabilization of the spinal column [25]. Indications for surgery generally include the presence of mechanical compression, spinal instability, or progressive neurological deterioration [18].

The choice of surgical technique is a critical component of management and depends on the underlying pathology. Anterior approaches, such as anterior cervical discectomy and fusion or corpectomy, are typically preferred when compressive pathology is located ventrally, including disc herniation, osteophytes, or ossification of the posterior longitudinal ligament. These techniques allow for direct decompression and restoration of cervical alignment. Posterior approaches, including laminectomy with or without fusion and laminoplasty, are more commonly employed in cases of multilevel stenosis or when compression extends across multiple segments. These techniques provide indirect decompression through posterior cord drift and may be advantageous in patients with preserved cervical lordosis [18,20]. Importantly, current evidence does not demonstrate a clear superiority of one approach over another, as outcomes are largely determined by appropriate patient selection and surgical indication rather than the approach itself [10,26].

A critical aspect of surgical management is the timing of intervention. Increasing evidence supports the concept of early decompression, typically within the first 24-72 hours following injury, as a means of limiting secondary injury processes such as ischemia, edema, and inflammatory cascades. Early surgical intervention has been associated with improved neurological recovery, particularly in upper extremity function, which is disproportionately affected in CCS [7,8,20]. However, the literature remains heterogeneous, and some studies report comparable outcomes between early and delayed surgery, suggesting that timing alone may not be the sole determinant of recovery [13].

When directly comparing conservative and surgical management, the available evidence suggests that both approaches can lead to meaningful neurological improvement, but in different patient populations [1,27]. Conservative treatment appears to be most effective in patients with mild deficits, absence of instability, and no significant cord compression, particularly when early clinical improvement is observed [23,24]. In contrast, surgical management demonstrates advantages in patients with identifiable structural pathology, progressive neurological decline, or more severe deficits. Early surgical intervention in these patients may accelerate recovery and improve functional outcomes, especially in terms of upper limb motor function [8,28].

To further clarify the clinical response observed following each treatment strategy, Table 2 summarizes the pretreatment clinical context and the post-treatment neurological or functional outcomes reported in the included studies. Only studies providing treatment-related outcome information were included in this focused comparison. Diagnostic studies, narrative discussions without separately extractable treatment outcomes, and clinical guidelines were not repeated in this table.

**Table 2:** Treatment-related outcomes reported in the included studies. ASIA, American Spinal Injury Association; CCS, central cord syndrome; JOA, Japanese Orthopaedic Association; SCI, spinal cord injury

Study	Pretreatment clinical context	Management	Follow-up outcome	Main interpretation
Schneider et al. (1954) [3]	Acute traumatic CCS with disproportionate upper-extremity weakness	Conservative treatment	Spontaneous neurological recovery was frequently observed, generally beginning in the lower extremities and progressing to the upper extremities	Established the historical basis for initial conservative management
Yelamarthy et al. (2019) [8]	Acute traumatic CCS	Early vs delayed surgical treatment	Surgical decompression was generally followed by neurological recovery; early surgery appeared safe and potentially beneficial, although the evidence was heterogeneous	Early surgery may be considered in appropriately selected patients
Jia et al. (2023) [9]	CCS without cervical fracture or dislocation	Different surgical decompression techniques	Significant postoperative neurological and functional improvement was observed with the evaluated surgical approaches	The surgical approach should be selected according to the location and extent of the compressive pathology
Zhou et al. (2022) [10]	Acute traumatic CCS	Anterior vs posterior surgery	Both approaches improved JOA and ASIA scores; no clear neurological superiority of either approach was demonstrated	Both approaches are effective when appropriately selected; the choice should be individualized
Chiu et al. (2020) [12]	CCS requiring surgical intervention	Early vs delayed surgery	Early surgery was associated with a shorter hospital stay, reduced hospital costs, and a more favorable discharge disposition	Earlier surgery may improve short-term hospital outcomes
Hosman et al. (2023) [13]	Acute traumatic SCI, including patients with CCS	Early vs delayed decompression	Earlier decompression was associated with superior neurological recovery in patients with incomplete SCI	Findings support early decompression when persistent surgically remediable compression is present
Nakajima et al. (2023) [20]	Cervical SCI without major bone injury	Contemporary conservative and surgical management	Patients with persistent spinal cord compression were more likely to benefit from surgery, whereas selected stable patients could be treated nonoperatively	MRI findings, neurological progression, and spinal stability should guide treatment
Ishida and Tominaga (2002) [23]	Mild CCS with predominantly upper-extremity deficits	Conservative management	Most patients demonstrated rapid neurological recovery, particularly during the first six weeks	Conservative treatment may be appropriate in patients with mild deficits and early improvement
Fong and Eismont (2007) [24]	Acute CCS of variable severity	Conservative vs surgical management	Neurological improvement was reported with both strategies; surgery could accelerate recovery in patients with persistent compression or a neurological plateau	Management should depend on spinal stability, compression, and the course of neurological recovery
Kato and Fehlings (2017) [25]	CCS without overt mechanical instability	Conservative vs surgical management	Surgical decompression was associated with improved neurological recovery when persistent spinal cord compression was present	Absence of instability alone does not exclude a potential indication for surgery
Ding et al. (2024) [26]	Cervical SCI treated surgically	Anterior vs posterior surgery	Both approaches produced neurological and functional improvement, without a significant overall difference between them	Surgical approach should be individualized rather than selected routinely
Chen et al. (2023) [29]	Cervical SCI without fracture or dislocation	Surgical vs conservative treatment	Surgical treatment produced greater improvement in neurological function and JOA scores than conservative treatment	Surgery may provide superior recovery in appropriately selected patients, although the study population was not limited exclusively to CCS

These findings demonstrate that improvement may occur following either conservative or surgical management, although the two approaches are generally used in different patient populations. Conservative treatment was associated with favorable recovery primarily in patients with mild, stable, and early-improving deficits without instability or persistent surgically remediable compression. Surgical treatment was associated with neurological improvement in patients with persistent compression, more severe deficits, instability, or neurological deterioration. Therefore, the findings should not be interpreted as a direct demonstration of the universal superiority of either strategy.

It is important to emphasize that the apparent superiority of one approach over the other in the literature is often influenced by selection bias, as patients undergoing surgery typically present with more severe pathology. Consequently, direct comparisons between conservative and surgical cohorts must be interpreted with caution. The absence of large-scale randomized controlled trials further limits the strength of the available evidence and contributes to ongoing controversy in clinical practice [7,27,30].

Current clinical recommendations, derived from broader spinal cord injury guidelines, support an individualized approach to management. Organizations such as the American Association of Neurological Surgeons and the Congress of Neurological Surgeons advocate for early surgical decompression in patients with ongoing spinal cord compression or neurological deterioration. Similarly, AO Spine recommendations emphasize the potential benefits of early intervention in traumatic spinal cord injury, including CCS, when clinically indicated. Nevertheless, the lack of CCS-specific high-level evidence underscores the need for clinical judgment in each case [7,30]. Representative imaging findings in patients with traumatic CCS are shown in Figure 2.

**Figure 2:**
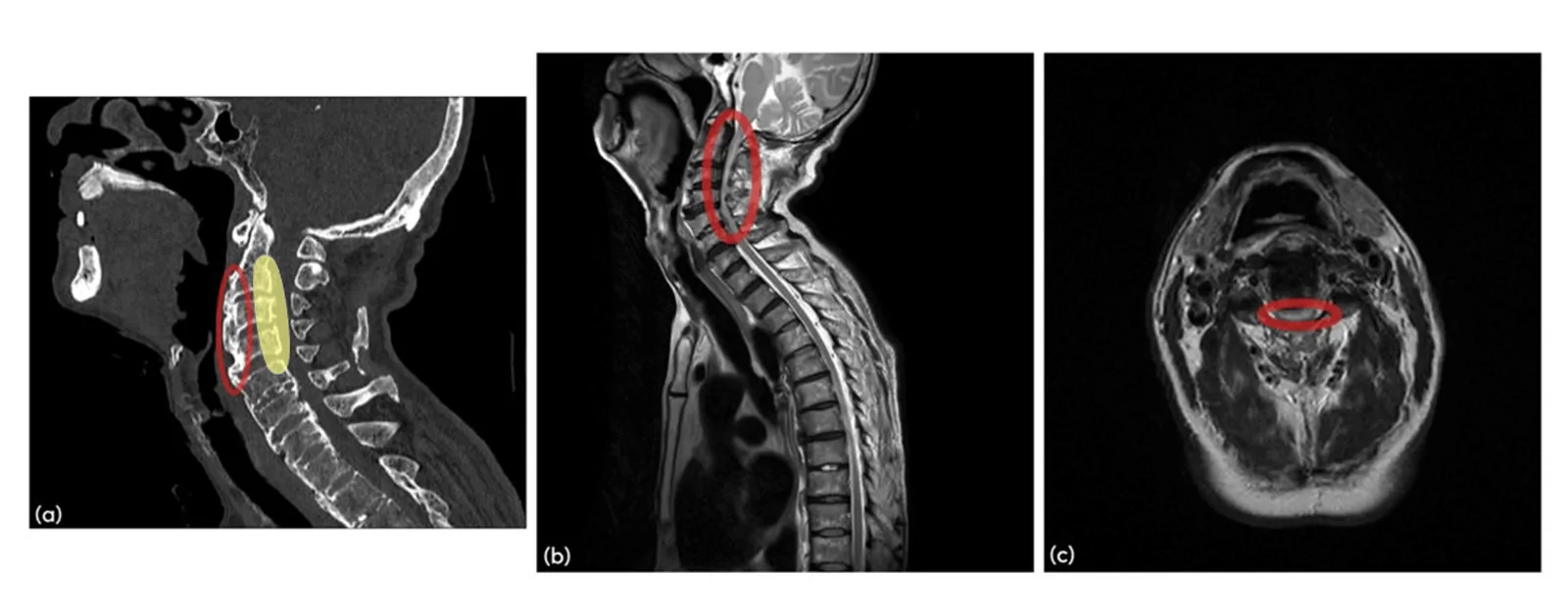
CT and MRI findings in traumatic CCS. (A) Sagittal CT showing preserved cervical alignment without fracture. Multilevel degenerative changes and ligamentous ossification from C2 to C7 cause significant spinal canal stenosis. The red outline indicates anterior ligamentous hyperostosis, and the yellow highlight indicates ossification of the posterior longitudinal ligament. (B) Sagittal T2-weighted MRI showing central spinal cord edema extending from C2 to C6 (red outline), with multilevel canal stenosis that is most severe at C4-C5. (C) Axial T2-weighted MRI showing central spinal cord edema (red outline). CCS, central cord syndrome Image source: Guan (2022) [31], used under the Creative Commons Attribution-NonCommercial-ShareAlike 3.0 Unported License (CC BY-NC-SA 3.0). Colored annotations were added by the authors.

Several prognostic factors play a key role in guiding treatment decisions. Initial neurological status, particularly as assessed by the ASIA Impairment Scale [32], is consistently identified as the strongest predictor of outcome. Patients with less severe deficits are more likely to recover regardless of treatment modality. Age also plays an important role, with younger patients demonstrating better functional recovery. Imaging findings, including the extent of spinal cord signal changes on MRI and the presence of preexisting spinal stenosis, further contribute to prognostic assessment and treatment planning [7,8,29].

In summary, the management of CCS requires a nuanced and individualized approach. Conservative and surgical strategies are not mutually exclusive but rather complementary, each playing a role depending on the clinical context. The integration of clinical evaluation, imaging findings, and patient-specific factors is essential in guiding optimal treatment and improving neurological and functional outcomes (Table 3) [1,27]. 

**Table 3:** Summary of the included studies. ASIA, American Spinal Injury Association; CCS, central cord syndrome; JOA, Japanese Orthopaedic Association; SCI, spinal cord injury

Study	Study design	Population	Intervention/comparison	Main outcomes	Main findings
Avila and Hurlbert (2021) [1]	Narrative review	Traumatic CCS	Contemporary management	Functional recovery	Recommended individualized management based on MRI findings, spinal stability, and neurological examination.
Engel-Haber et al. (2023) [2]	Narrative review	CCS	Definition and classification	Diagnostic consistency	Highlighted variability in CCS definitions and emphasized the need for standardized diagnostic criteria.
Schneider et al. (1954) [3]	Case series	Acute traumatic CCS	Conservative treatment	Neurological recovery	First description of CCS. Recommended initial conservative management because spontaneous neurological recovery was frequently observed.
Brooks (2017) [4]	Narrative review	CCS	Review of treatment strategies	Clinical outcomes	Summarized indications for conservative and surgical treatment according to neurological status and radiological findings.
Charles et al. (2024) [5]	Narrative review	CCS	Current concepts	Functional outcomes	Modern evidence supports individualized surgical decision-making rather than routine conservative treatment.
Yelamarthy et al. (2019) [8]	Systematic review	Acute traumatic CCS	Early vs delayed surgery	Neurological recovery	Early decompression appears safe and may improve neurological recovery, although evidence remains limited.
Jia et al. (2023) [9]	Retrospective comparative study	CCS without fracture/dislocation	Different surgical techniques	Functional recovery	Both anterior and posterior surgery significantly improved neurological function; approach should depend on pathology.
Zhou et al. (2022) [10]	Comparative cohort study	Acute traumatic CCS	Anterior vs posterior surgery	JOA, ASIA scores	Similar neurological improvement with both approaches; anterior surgery associated with less blood loss and shorter hospitalization.
Chiu et al. (2020) [12]	Nationwide retrospective cohort	CCS	Early vs delayed surgery	Length of stay, discharge destination, hospital cost	Early surgery significantly reduced hospital stay and healthcare costs while improving discharge disposition.
Hosman et al. (2023) [13]	Systematic review	Acute traumatic SCI (including CCS)	Early vs delayed decompression	Neurological recovery	Early (<24 hours) decompression was associated with superior neurological recovery in patients with incomplete SCI.
Pouw et al. (2010) [15]	Diagnostic study	Patients with traumatic CCS	Diagnostic criteria	Diagnostic accuracy	Proposed standardized diagnostic criteria to improve consistency in CCS diagnosis.
Nakajima et al. (2023) [20]	Narrative review	Cervical SCI without major bone injury	Contemporary management	Clinical outcomes	MRI-based assessment is essential; early surgery recommended in patients with persistent spinal cord compression.
Ishida and Tominaga (2002) [23]	Retrospective cohort	Mild CCS with isolated upper extremity deficits	Conservative management	Motor recovery	Most patients recovered rapidly within the first six weeks; conservative treatment appropriate in carefully selected patients.
Fong and Eismont (2007) [24]	Narrative review	Acute CCS	Conservative vs surgical management	Neurological recovery	Surgical decompression may accelerate recovery when persistent spinal cord compression or neurological plateau is present.
Kato and Fehlings (2017) [25]	Narrative review	CCS without instability	Surgery vs conservative treatment	Neurological recovery	Surgical decompression is recommended when persistent spinal cord compression is demonstrated despite the absence of instability.
Ding et al. (2024) [26]	Meta-analysis	Cervical SCI	Anterior vs posterior surgery	Functional recovery	No significant neurological difference between surgical approaches; treatment should be individualized.
Fehlings and Gray (2011) [27]	Expert review/book chapter	Elderly CCS	Surgical management	Neurological outcome	Discussed advantages of decompression in elderly patients with cervical stenosis and CCS.
Camino-Willhuber et al. (2026) [28]	Clinical practice recommendations	Subaxial cervical spine injuries	Evidence-based treatment recommendations	Surgical indications	Recommends surgical stabilization in unstable injuries according to fracture morphology and neurological status.
Chen et al. (2023) [29]	Meta-analysis	Cervical SCI without fracture/dislocation	Surgery vs conservative treatment	Neurological recovery	Surgical treatment demonstrated superior neurological recovery compared with conservative treatment in appropriately selected patients
American College of Surgeons (2022) [30]	Clinical practice guideline	Cervical spine trauma	Best practice recommendations	Acute management	Recommends CT as first-line imaging and surgery for instability or neurological deficit following multidisciplinary assessment.

Limitations

This study has several limitations that should be acknowledged. First, the included studies demonstrated significant heterogeneity in terms of study design, patient populations, definitions of CCS, and outcome measures, which limits the ability to perform direct comparisons and draw definitive conclusions. Second, the majority of the available evidence is derived from retrospective and observational studies, while high-quality randomized controlled trials remain scarce. This limitation reduces the overall strength of the evidence and may affect the generalizability of the findings. Furthermore, potential selection bias should be considered, as patients undergoing surgical management often present with more severe neurological deficits or radiological findings, which may influence the perceived superiority of surgical intervention in certain cohorts.

Finally, variability in surgical techniques, timing of intervention, and rehabilitation protocols across studies introduces additional confounding factors that may impact clinical outcomes. As this scoping review does not include an original patient cohort, paired pre- and post-treatment imaging was not available. The imaging presented in Figure 2 is illustrative and should not be interpreted as comparative evidence of treatment effectiveness. Future research should focus on standardized methodologies and prospective comparative studies to better define optimal treatment strategies.

## Conclusions

CCS represents a complex and heterogeneous clinical entity that requires a carefully individualized management approach. Although conservative treatment remains a valid and effective option in patients without spinal instability or significant spinal cord compression, particularly in the presence of mild and improving neurological deficits, it is increasingly evident that this approach is not universally applicable.

The findings of the present review support a more selective and dynamic treatment strategy, in which surgical intervention plays a central role in appropriately chosen patients. In cases of mechanical compression, spinal instability, or progressive neurological deterioration, surgical decompression, particularly when performed early, appears to facilitate improved neurological and functional recovery, with a notable impact on upper extremity function. Rather than representing competing strategies, conservative and surgical management should be viewed as complementary approaches within a patient-centered decision-making framework. The integration of clinical assessment, imaging findings, and prognostic indicators is essential in guiding optimal treatment selection. Despite advances in understanding and management, the current evidence remains limited by heterogeneity and the lack of high-quality randomized studies. Future research should focus on standardized diagnostic criteria, prospective comparative studies, and long-term functional outcomes in order to better define treatment algorithms and improve patient care.
